# Health-Related Quality of Life and Fatigue in Patients with Osteogenesis Imperfecta

**DOI:** 10.3390/healthcare14121620

**Published:** 2026-06-09

**Authors:** Filiz Mercan Sarıdaş, Erhan Hocaoğlu, Müge Yaşar, Kadircan Karatoprak, Güven Özkaya, Soner Cander, Erdinç Ertürk, Canan Ersoy, Özen Öz Gül

**Affiliations:** 1 Division of Endocrinology and Metabolism, Department of Internal Medicine, Faculty of Medicine, Bursa Uludağ University, 16059 Bursa, Turkey; 2 Department of Biostatistics, Faculty of Medicine, Bursa Uludağ University, 16059 Bursa, Turkey

**Keywords:** osteogenesis imperfecta, health-related quality of life, fatigue, bisphosphonate, fracture

## Abstract

**Objective**: This study aimed to assess health-related quality of life (HRQoL) and fatigue in patients with osteogenesis imperfecta (OI) and to compare these outcomes with those of healthy controls. In addition, the associations between fatigue and the physical and mental components of HRQoL, as well as related clinical factors, were examined. **Method**: Between June 2024 and June 2025, 27 adults with OI were enrolled and compared with 27 healthy controls. Fatigue was assessed with the FSS and HRQoL with the SF-36, including PCS and MCS scores. We evaluated factors associated with the PCS and MCS and examined the associations between the PCS/MCS and fatigue. **Results**: PCS scores were significantly lower in patients with OI types III and IV compared with controls, whereas no difference was observed between type I and controls. No significant differences were found in MCS scores or mental health domains among the groups. Fatigue levels did not differ significantly among the groups. Height was positively correlated with the PCS, while the number of fractures showed a moderate negative correlation. Lower PCS scores were observed in patients with lower extremity deformities and a history of orthopedic surgery. Alendronate use and longer treatment duration showed associations with higher PCS scores. In addition, a significant negative correlation was found between the MCS and fatigue severity. **Conclusions**: Adults with OI showed impaired physical functioning compared with controls, with relatively preserved mental functioning and similar fatigue levels. Physical functioning was associated with height, fracture burden, orthopedic surgery, and alendronate use, while fatigue was associated with mental functioning, underscoring the need for patient-centered care addressing both skeletal and psychosocial outcomes.

## 1. Introduction

Osteogenesis imperfecta (OI) is a connective tissue disorder that arises from mutations in the COL1A1 and COL1A2 genes, which encode type 1 collagen. The disorder is typically characterized by autosomal dominant inheritance. The prevalence of the disease is reported to be between 1 and 2 cases in 10,000 individuals. The disease is traditionally categorized into four groups according to the Sillence classification (type I: mild, type II: neonatal lethal, type III: severe, and type IV: moderate) [1,2]. Although the revised classification identifies 18 subtypes, clinical practice still largely relies on the Sillence types [3,4].

OI is a heterogeneous disorder with varying clinical features and severity. Type I OI is characterized by fractures that typically occur during childhood and adolescence, often resulting in normal height and no deformity. These patients are typically capable of independent movement. In contrast, individuals diagnosed with type III OI are typically identified at birth and are distinguished by progressive lower extremity and spinal deformities. Many patients in this category ultimately become wheelchair-dependent and, consequently, unable to move independently [5]. Patients may present with musculoskeletal manifestations such as dentinogenesis imperfecta, macrocephaly, pectus carinatum, pectus excavatum, and joint hypermobility. In addition, various extraskeletal manifestations may occur, including blue–gray sclera, hearing impairment, easy bruising, respiratory disorders, and cardiovascular abnormalities [6,7]. The prevalence of musculoskeletal impairments and related deformities has been shown to have a substantial negative impact on health-related quality of life (HRQoL) in affected individuals. Although there is no curative treatment for OI, pharmacological therapies aimed at increasing bone mineral density and orthopedic surgical interventions designed to treat fractures and prevent deformities constitute the mainstays of management. In addition to these treatments, there is an increasing need for a comprehensive approach that focuses on improving quality of life, maintaining physical functioning and mental health, and promoting independence [8,9]. The current literature indicates that a substantial proportion of patients with OI experience significant fatigue. In adults with OI, fatigue may arise from multiple and interacting mechanisms, including increased energy expenditure during ambulation due to skeletal deformities and altered biomechanics, muscle weakness, chronic pain, recurrent fractures, reduced physical conditioning, and the psychological burden of living with a lifelong skeletal disorder. Although fatigue can negatively affect HRQoL, it represents a distinct patient-reported symptom rather than merely a subdomain of quality of life. Fatigue may interfere with motivation, exercise, daily responsibilities, work, family life, and social participation and therefore provides complementary information to generic HRQoL measures [10,11,12,13].

Evidence regarding differences in fatigue and HRQoL in patients with OI, particularly in comparison with healthy controls, remains limited, particularly due to small sample sizes, heterogeneous populations, and the lack of well-matched control groups. Therefore, this study aimed to evaluate HRQoL and fatigue in patients with OI types I, III, and IV and to compare these outcomes with those of healthy individuals. We hypothesized that patients with OI would have a lower quality of life than controls. Additionally, this study aimed to explore the associations between fatigue, HRQoL components, and clinical factors such as bone mineral density, fracture history, and treatments.

## 2. Materials

### 2.1. Patient Selection

A total of 36 patients followed at our center were assessed for eligibility; nine were excluded due to an inability to be contacted or refusal to participate, and the remaining 27 patients were included between June 2024 and June 2025. A healthy control group was consecutively selected during the same period and sex-matched (1:1) to the patient group, while formal age matching was not performed because of feasibility constraints related to the limited number of eligible adult patients with this rare disease. The controls consisted of individuals without OI, aged ≥18 years, with no chronic disease, not using any medications, and with no neurological or psychiatric disorders that could affect cognition or mood.

### 2.2. Clinical Assessments

According to the Sillence classification, 15 patients were classified as type I OI, 5 as type III, and 7 as type IV. Written informed consent was obtained from all patients and healthy controls.

All patients underwent a standardized interview and physical examination. Demographic and clinical data were recorded, including anthropometric measurements, mobilization status, education, employment and marital status, smoking habits, and family history, as well as OI-related physical findings. Medical records and patient reports were reviewed to obtain genetic results, dual-energy X-ray absorptiometry (DXA)-based bone densitometry measures, fracture history (number and age at first fracture), orthopedic surgery history, and past/current treatments with their durations; the most recent DXA values were recorded.

Health-related quality of life was assessed using the Turkish-validated 36-item Short Form Health Survey (SF-36), which includes eight domains [14]. Physical functioning, role physical, bodily pain, and general health contribute to the Physical Component Summary (PCS), while vitality, role emotional, social functioning, and mental health contribute to the Mental Component Summary (MCS). Domain scores range from 0 to 100, with higher scores indicating better HRQoL. Since Turkey-specific norm-based PCS/MCS coefficients are not available, the PCS and MCS were calculated using the standard norm-based scoring method with coefficients derived from the US general population. In addition, summary scores were recalculated using the Hungarian population norm-based scoring parameters published in 2025 to assess their robustness and cultural relevance [15,16]. The Fatigue Severity Scale (FSS) was used to assess fatigue severity as a symptom-specific outcome distinct from generic HRQoL. The FSS consists of nine items evaluating the impact of fatigue on motivation, exercise, physical functioning, daily responsibilities, work, family, and social life. It is a standardized and widely used measure of fatigue, including in rare-disease settings. Fatigue severity was calculated as the mean score across the nine items, with higher scores indicating greater fatigue. For categorical analyses, participants were classified as having normal fatigue (FSS < 4) or high fatigue (FSS ≥ 4).

### 2.3. Statistical Analysis

The normality of the data was assessed using the Shapiro–Wilk test. In order to compare the SF-36 and FSS scores between the OI subtypes and the control group, one-way ANOVA was employed for variables that demonstrated a normal distribution, whilst the Kruskal–Wallis test was utilized for variables that did not demonstrate a normal distribution. Comparisons of categorical variables between OI subtypes were performed using the chi-square test or Fisher’s exact test, and Monte Carlo correction was applied when appropriate (e.g., for tables larger than 2 × 2 or when expected cell counts were low). In order to evaluate the relationships between the PCS and continuous variables, correlation analyses were performed. The Pearson correlation coefficient was employed for variables exhibiting a normal distribution, while the Spearman correlation coefficient was utilized for variables that did not demonstrate a normal distribution. To compare the PCS and MCS scores with binary categorical variables, independent samples *t*-tests were employed for variables exhibiting a normal distribution, while Mann–Whitney U tests were utilized for variables failing to demonstrate a normal distribution. Due to the limited sample size, multivariable regression analyses were not performed.

## 3. Results

This study comprised 27 patients with osteogenesis imperfecta (15 patients with type I, five with type III, and seven with type IV) and a healthy control group of 27 individuals of similar age and gender. The median age in the OI group was 36 (18–66), and in the control group, it was 38 (21–55). The groups’ age distributions were comparable. In both groups, 14 patients (51.9%) were female.

Concerning height, type III and type IV subjects were found to be significantly shorter than the control group, with type III subjects being significantly shorter than type I subjects (*p* < 0.001, *p* = 0.001, and *p* = 0.004, respectively). The BMI was found to be significantly higher in type III compared to type I and the control group (29.3 vs. 23.8, *p* = 0.002, and 29.3 vs. 26.4, *p* = 0.034, respectively). Upon BMI categorization, no statistically significant differences were observed between the groups (*p* = 0.123). Educational attainment, employment status, and smoking habits differed across the groups and are summarized in Table 1. Genetic results were available for 21 patients. The genetic analyses revealed the presence of a COL1A1 mutation in 11 patients, a COL1A2 mutation in seven patients, and a LEPRE1 gene mutation in one patient. Additionally, a PLS3 gene mutation was identified in one patient. Notably, in one patient for whom genetic analysis was conducted, no pathogenic variant of OI was detected. However, the patient exhibited physical examination findings consistent with OI. A COL27A1 mutation was detected in this patient. This mutation has been reported in the literature to be associated with the development of pectus excavatum in two siblings with OI who also carried a COL1A1 mutation; however, our patient’s physical examination did not reveal pectus excavatum [17].

An examination of the patients’ mobilization status revealed that 93.3% of those with type I and all of those with type IV were fully mobile. However, it was noted that one patient with type I OI and one patient with type III OI were using crutches. In the OI type III group, the remaining four patients were wheelchair-dependent. Therefore, patients with type III OI represented a clinically more severe and functionally distinct subgroup compared with the predominantly ambulatory type I and type IV groups. All patients with type III OI had lower extremity deformities. The present study found that 86.7% of type I cases, 80% of type III cases, and 57.1% of type IV cases exhibited blue or gray sclera. Dentinogenesis imperfecta was observed in all cases of type III, 66.7% of type I, and 57.1% of type IV. A family history was documented in 80% of patients with types I and III and in 71.4% of patients with type IV. The patients’ physical examination findings are detailed in Table 2.

No statistically significant difference was found in the groups’ bone densitometry results in terms of total lumbar and total femur BMD. The number of fractures was higher for type III, as expected, with more than 10 fractures found in 80% of type III cases and 14.3% of type IV cases. A subsequent analysis involved a comparison of the mean age at first fracture across the type I, type III, and type IV groups. While the mean age at first fracture was found to be lower in the type III group (8 (1–35) years), this difference was not found to be statistically significant (*p* = 0.058). The study revealed no statistically significant differences between the groups with respect to patients with a history of orthopedic surgery. Orthopedic surgical history was further evaluated according to OI subtype. Among 16 patients with a history of orthopedic surgery, a total of 11 procedures were recorded in six patients with type I OI, 38 procedures in five patients with type III OI, and nine procedures in five patients with type IV OI. Surgery was performed only for fracture-related indications in eight patients, only for deformity-corrective/reconstructive indications in two patients, and for both fracture-related and deformity-corrective/reconstructive indications in five patients. Regarding timing, surgery was performed only during childhood in eight patients, only during adulthood in four patients, and during both childhood and adulthood in four patients. In one patient, the surgical indication could not be clearly determined from the available records. The bone results for the cohort are displayed in Table 3.

Furthermore, the study revealed that 80% of type III OI patients received pamidronate treatment, in contrast to the 26.7% of type I patients who did so (*p* = 0.013). There was no statistically significant difference between the groups in terms of the rates of zoledronic acid and alendronate intake. A significant finding emerged from the examination of treatment durations, wherein the duration of pamidronate administration was found to be considerably prolonged in type III, with a duration of 102 months. This is demonstrated in Table 4, where treatment histories and durations are presented.

**Figure 1:**
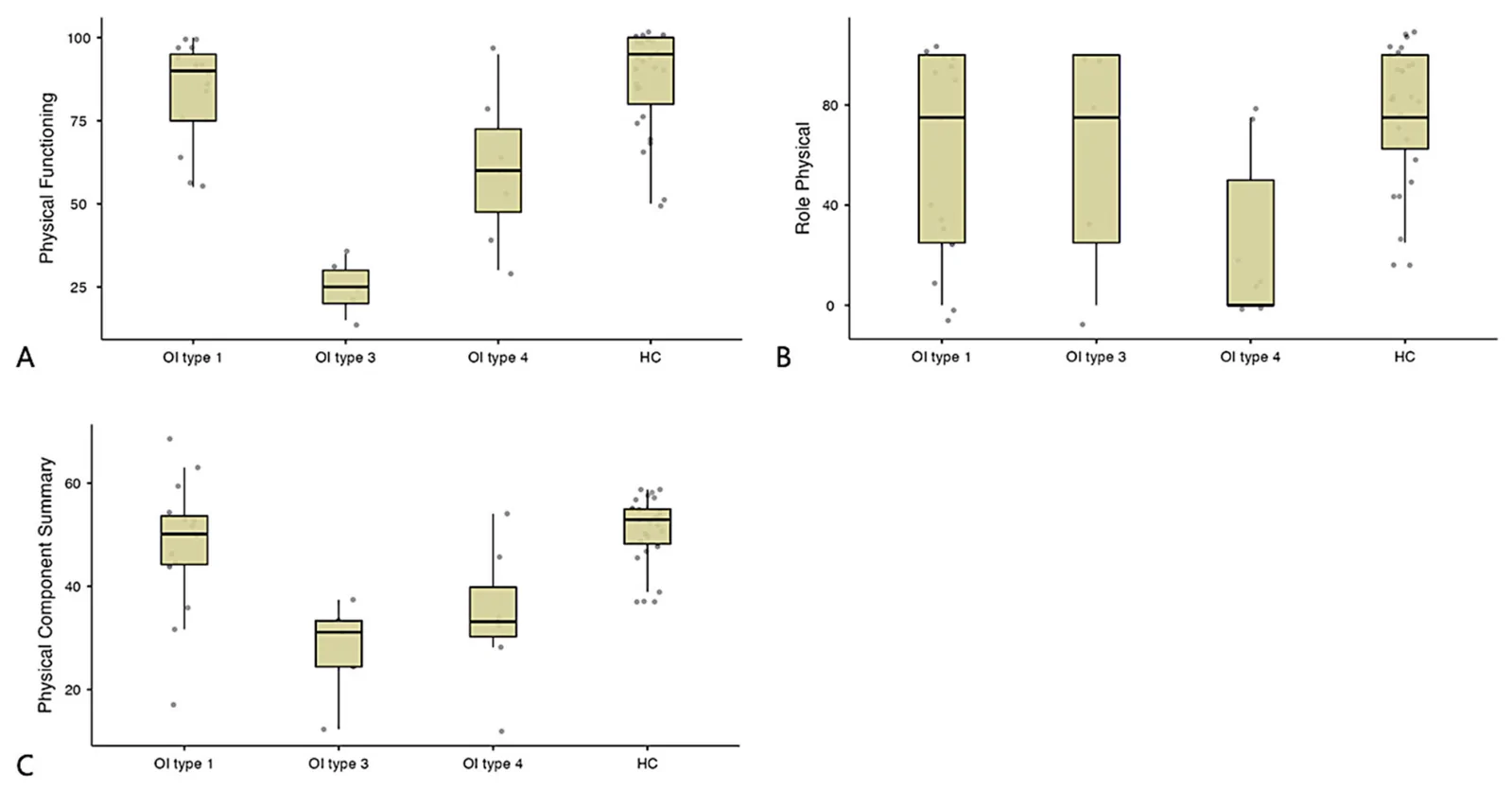
Physical domain scores on SF-36 and Physical Component Summary in patients with osteogenesis imperfecta and healthy controls. Data are presented as median values with interquartile ranges. OI: osteogenesis imperfecta; HC: healthy control group. (**A**) Physical function was lowest in type III, lower in both type III and type IV compared to HC (*p* < 0.001 and *p* = 0.006, respectively), and significantly lower in type III compared to type I (*p* = 0.001). (**B**) Physical role limitations were lower in type IV compared to type I and HC (*p* = 0.032 and *p* = 0.002). (**C**) Physical component summary scores were found to be significantly lower in types III and IV compared to HC (*p* < 0.001 and *p* = 0.003, respectively); however, no statistically significant difference was found between type I and HC (*p* = 0.373). The dots represent the values for each patient, whilst each box and the line within it represent the median (min-max) values.

An analysis of the SF-36 parameters revealed that physical function (PF) was lowest in type III OI (25), lower in both type III and type IV compared to the control group (*p* < 0.001 and *p* = 0.006, respectively), and significantly lower in type III compared to type I (*p* = 0.001). When physical role limitations (RP) were taken into account, a substantial decrease was identified in type IV compared to type I and the control group (*p* = 0.032 and *p* = 0.002). Although the omnibus one-way ANOVA indicated a significant between-group difference in SF-36 general health (GH) scores, post hoc pairwise comparisons did not reveal any significant differences. No significant differences were observed between the groups with respect to body pain (BP). PCS scores were found to be significantly lower in types III and IV compared to the control group (*p* < 0.001 and *p* = 0.003, respectively); however, no statistically significant difference was found between type I and the control group (*p* = 0.373). No significant differences were identified between the groups in any of the mental health parameters or in the MCS. Elevated FSS results were observed in type IV OI (5.77), but these did not yield a statistically significant difference. The prevalence of fatigue among patients with OI type IV was found to be 71.4%, while the lowest percentage was observed in OI type III, at 20% (*p* = 0.343) (Figure 1, Table 5).

### Assessment of Factors Affecting PCS

a-Demographic data and bone parameters

**Figure 2:**
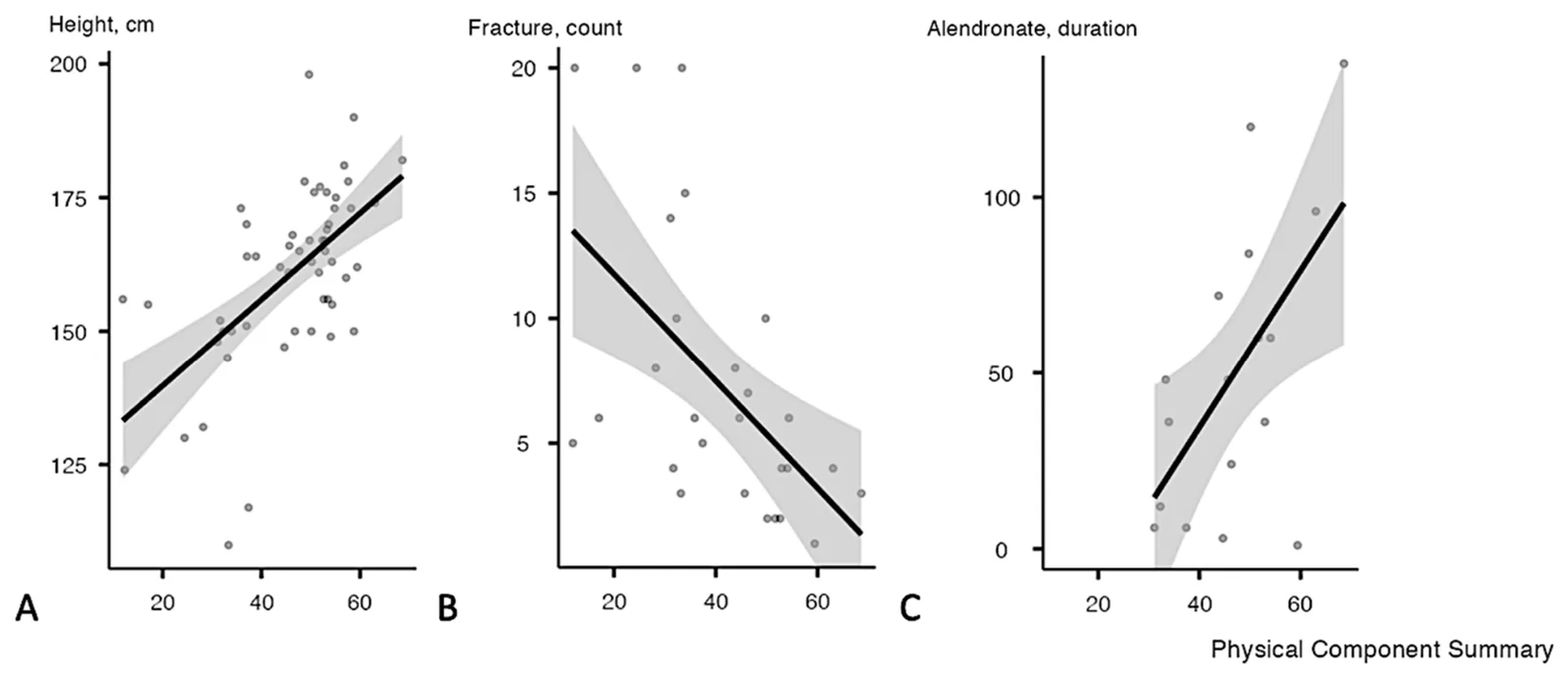
Associations between Physical Component Summary score and selected clinical variables. Scatter plots illustrating relationships between PCS score and (**A**) height, (**B**) number of fractures, and (**C**) duration of alendronate use. (**A**) Positive correlation between height and PCS (r = 0.599, *p* < 0.001). (**B**) Negative correlation between fracture count and PCS (r = –0.557, *p* = 0.003). (**C**) Positive correlation between alendronate duration and PCS (r = 0.592, *p* = 0.012).

Pearson correlation analysis demonstrated a strong positive correlation between height and PCS scores (r = 0.599, *p* < 0.001) (Figure 2). No significant association was found between BMI and the PCS (*p* = 0.288), and PCS scores did not differ between smokers and non-smokers (*p* = 0.880). Lumbar spine BMD showed a positive but non-significant correlation with the PCS (r = 0.343, *p* = 0.087). Fracture count was moderately and negatively correlated with the PCS (r = –0.557, *p* = 0.003) (Figure 2). No significant association was observed between age at first fracture and the PCS (*p* = 0.107). PCS scores were significantly lower in patients with lower extremity deformities and in those with a history of orthopedic surgery, as compared with those without (*p* = 0.031 and *p* = 0.038, respectively).

b-Treatments

No significant difference in PCS scores was observed between patients receiving pamidronate and those not receiving it (*p* = 0.785), and no correlation was found between the pamidronate treatment duration and PCS scores (*p* = 0.075). PCS scores were higher in patients receiving alendronate than in those not receiving alendronate (46.95 ± 11.00 vs. 30.15 ± 14.89, *p* = 0.002), and a positive correlation was observed between the alendronate treatment duration and PCS scores (r = 0.592, *p* = 0.012) (Figure 2). Patients not receiving zoledronic acid had higher PCS scores compared with those receiving zoledronic acid (*p* = 0.004). No significant correlation was observed between the total bisphosphonate treatment duration and PCS scores (*p* = 0.083). These treatment-related findings were exploratory and were not adjusted for disease severity or indication for treatment.

c-MCS and FSS

No significant correlation was observed between PCS and MCS scores (rho = –0.175, *p* = 0.205). PCS scores showed a weak, non-significant negative correlation with FSS scores (rho = –0.246, *p* = 0.073). Educational level was not significantly associated with MCS scores (*p* = 0.089). A moderate negative correlation was identified between the MCS and fatigue severity, with lower MCS scores associated with higher FSS scores (rho = –0.441, *p* < 0.001).

## 4. Discussion

The present study sought to establish a comparison between the SF-36 and FSS scores of patients diagnosed with OI and those of a healthy control group. In the extant literature, the quality of life of adult OI patients has been predominantly compared with population norm data. Nevertheless, there is a paucity of studies that make such comparisons with a healthy control group. Our findings demonstrate that patients with OI exhibit significantly lower physical HRQoL compared with healthy controls, while mental health components remain largely preserved. The extant literature generally demonstrates that physical functions are affected and mental functions are preserved, a finding that is consistent with the results of the present study. However, some studies have reported that mental components may also be affected to a lesser extent, and that parameters such as vitality, social functioning, and emotional role limitations, which are represented by mental component subscores, may also be affected [9,13,18,19,20]. In contrast, fatigue severity in our cohort was comparable to that in the control group, a finding that is supported by some but not all previous studies [12,13,21].

The nature of the disease OI is such that it can result in several physical health complications. These include fractures, deformities, musculoskeletal pain, and associated mobility limitations. These factors can significantly impact patients’ day-to-day activities, including walking, running, and climbing stairs. This, in turn, can lead to limitations in physical function and physical role performance. In the present study, the finding that physical function scores were significantly lower in adult OI patients compared to healthy controls is consistent with these clinical characteristics.

Physical function was lower in patients with type III and type IV OI than in healthy controls, consistent with the greater musculoskeletal burden of these subtypes. However, these differences should be interpreted with caution, particularly for type III OI, as adults in this subgroup were largely wheelchair-dependent and, therefore, functionally distinct from both healthy controls and ambulatory patients with OI. Thus, the lower physical HRQoL observed in type III OI likely reflects the combined effects of disease severity, skeletal deformity, and impaired mobility and should be considered descriptive of the adult OI clinical spectrum, rather than a direct comparison between functionally comparable groups. The significantly lower physical function scores in type III compared with type I OI further suggest that the physical impact of OI is heterogeneous across subtypes and increases with clinical severity [9,22]. Adult mobility in severe OI may also be influenced by childhood orthopedic management. In our cohort, all patients with type III OI had lower extremity deformities, and most were wheelchair-dependent in adulthood; however, detailed childhood surgical data were limited, and the impact of early orthopedic interventions on adult mobility could not be assessed.

Despite all these physical limitations, patients with osteogenesis imperfecta generally have normal intelligence [23,24]. Physical impairment should not be interpreted as a limitation of intellectual potential, and patients with OI, including those with severe phenotypes, should be supported to attend school, continue education, and participate in social life according to their cognitive capacity and individual functional needs. Therefore, multidisciplinary care should not focus solely on fracture prevention, deformity correction, and mobility but should also aim to preserve educational access, independence, and social participation [8,25].

In the present study, no significant differences were observed in the mental health components of the SF-36 between patients with OI and healthy controls. This finding should be interpreted cautiously, as disease-specific psychological instruments and direct measures of coping strategies, adaptation, or response shift were not included. Therefore, preserved mental health scores cannot be attributed to these mechanisms with certainty. Nevertheless, the lifelong nature of OI, which often begins in childhood or at birth, may allow some individuals to adapt over time to the physical consequences of the disease. Concepts such as response shift and the disability paradox may partly explain why mental health scores do not necessarily parallel physical impairment, although these explanations remain hypothesis-generating. Furthermore, the regular monitoring of patients at major centers, the provision of family support, and the facilitation of rehabilitation processes may contribute to the preservation of mental health [22,26]. Further qualitative and longitudinal studies are needed to clarify how coping, adaptation, family support, and rehabilitation influence mental well-being in adults with OI.

Although fatigue severity did not differ significantly among the groups, higher FSS scores in type IV OI and lower scores in type III OI may reflect differences in mobility and daily activity. Ambulatory patients with type IV OI may perceive greater fatigue due to increased energy expenditure related to skeletal deformities, altered biomechanics, muscle weakness, and pain. In contrast, mostly wheelchair-dependent patients with type III OI may have lower daily energy demands or may have adapted their activities to their physical limitations. Thus, fatigue in OI may reflect the interaction among mobility, activity level, pain, and psychological adaptation, rather than physical disability alone. Previous studies have similarly reported no significant differences in total FSS scores among OI subtypes, although higher scores in selected FSS items were observed in type IV OI compared with types I and III [13].

The current study corroborates the findings of Matshusita et al., demonstrating a positive correlation between height and the PCS [27]. This relationship suggests that taller individuals tend to have superior physical health. As the severity of the disease increases, the short stature that is characteristic of OI becomes more pronounced [11]. These findings suggest that height may serve as both an indicator of disease severity and an important clinical parameter influencing PCS scores. However, the same relationship has not been demonstrated with BMI, and no other study in the literature has evaluated BMI from this perspective. These findings suggest that height, rather than body composition, may be a more relevant clinical indicator of physical functioning in adults with OI. The present study revealed no statistically significant differences between smokers and non-smokers with regard to the PCS. This may be attributable to the limited number and heterogeneity of the patients. To the best of our knowledge, there are no other studies in the literature that have evaluated this parameter in terms of physical function and quality of life with OI.

The present study found no correlation between PCS and MCS scores. While this may seem confusing at first glance, a review of the literature shows that physical function is generally worse in OI patients, while mental components are often preserved and sometimes even better than those in the general population [9,20,28]. Research conducted on the development of the SF-36 and analogous quality-of-life scales has indicated that the PCS and MCS demonstrate relative independence from each other, and that their low correlation is a distinguishing feature of these scores [15,29]. Murali and colleagues also obtained results similar to those obtained in this study [30].

In the present study, no correlation was found between FSS scores and the PCS, while a negative correlation was found with MCS scores. This finding suggests that the perception of fatigue in patients with OI may be more closely related to mental well-being than to the physical function level. A decline in mental health parameters may affect the individual’s motivation and stress coping level, thereby increasing fatigue perception. These findings underscore the necessity of incorporating psychosocial factors into the assessment and management of fatigue in patients suffering from OI. In the literature, Behanova et al. found that FSS scores were negatively correlated with both the PCS and MCS [31].

To date, few studies have directly examined the relationship between bone mineral density and quality of life in adults with OI [30]. Consistent with previous findings, we observed no association between lumbar spine BMD and PCS scores. This suggests that BMD alone does not adequately reflect functional status in OI. Physical functioning is influenced by multiple factors, including pain, deformities, fracture sequelae, muscle strength, and mobility limitations. In addition, spinal deformities common in OI may affect the accuracy of lumbar DXA measurements.

Fracture burden was negatively correlated with the PCS, and lower PCS scores were observed in patients with lower extremity deformities and a history of orthopedic surgery. These findings are consistent with those of Matsushita et al., who reported associations between lower extremity fractures, lower extremity orthopedic surgery, and the PCS [27]. Importantly, orthopedic surgery in OI should not be interpreted as a uniform intervention. In milder ambulatory phenotypes, surgery may mainly involve fracture management, whereas in severe phenotypes such as type III OI, surgery is more often related to deformity correction, reconstruction, and functional alignment. In our cohort, patients with type III OI had a substantially higher number of surgical procedures than those with type I or type IV OI, supporting the greater cumulative orthopedic burden in this subgroup. Farsetti et al. also emphasized the importance of reconstructive procedures aimed at restoring alignment and limb function for preserving mobility in complex skeletal disorders [32]. Therefore, the association between orthopedic surgery history and lower PCS scores likely reflects disease severity, deformity burden, and long-term musculoskeletal involvement, rather than the effect of surgery itself.

Associations between bisphosphonate use and physical HRQoL should be interpreted with substantial caution. In our study, higher PCS scores were observed in patients receiving alendronate, whereas lower PCS scores were observed in those treated with zoledronic acid, and no significant association was found with pamidronate use. However, these treatment-related findings are exploratory and highly susceptible to confounding by indication, because treatment allocation in OI is influenced by the baseline disease severity, fracture burden, mobility status, timing of diagnosis, and clinical follow-up patterns. Patients with type III OI are typically diagnosed early and are more likely to receive anti-osteoporotic treatment, whereas adults with type I OI may be diagnosed later in life and may have delayed or less frequent treatment exposure. Therefore, the observed associations should not be interpreted as causal or as direct effects of specific bisphosphonate agents on HRQoL. Previous studies have reported mixed results regarding the impact of antiresorptive therapies on HRQoL, particularly when treatment is initiated in adulthood [27,33,34,35,36].

While Hald and colleagues found a correlation between educational level and the MCS in their study, the present study found no difference in MCS scores across different educational levels [18]. In Behanova et al.’s study, which evaluated quality of life in those with rare metabolic bone diseases, no significant disparities in HRQoL scores were observed across varying educational levels, a finding that aligns with the results of our study [31]. Beyond educational and psychosocial factors, patient-centered management in OI should also be considered within a long-term care framework. Adults with OI often require lifelong multidisciplinary follow-up, including orthopedic assessment, fracture and deformity management, pharmacological monitoring, rehabilitation, and repeated evaluation of functional outcomes. In rare skeletal diseases, continuity of clinical information is particularly important because care may involve multiple specialties and repeated interventions over many years. In this context, digital infrastructures may improve data traceability, security, interoperability, and multidisciplinary collaboration [37].

### Limitations and Strengths

This study has several limitations. The relatively small and heterogeneous sample, the lack of a prospective design, and the retrospective collection of certain variables (such as fracture number and age at first fracture) based on medical records and patient self-reports may have introduced recall bias and missing data. The control group was sex-matched but not formally age-matched; although the groups’ age distributions were comparable, residual bias cannot be excluded because HRQoL and fatigue may be influenced by age. In addition, the SF-36 and FSS are not disease-specific instruments for skeletal disorders, which represents another limitation. Since our study population is from Turkey, we calculated the PCS and MCS using normative coefficients recently published for the Hungarian population, as well as those for the US. This was done to ensure robustness and cultural relevance, and to assess whether the interpretation of the results remained consistent across different normative reference datasets.

Despite these limitations, our study has several strengths. To the best of our knowledge, this is the first study to evaluate the HRQoL in adult patients with OI in Turkey. Unlike many previous studies that relied on population norms and external reference values, our study included a healthy control group, which strengthens the validity of our comparisons. Furthermore, we examined the associations between quality-of-life outcomes and several clinical and lifestyle factors that have been rarely explored in the literature, including bone mineral density, smoking status, and treatment types and durations.

## 5. Conclusions

In adults with OI, physical functioning was impaired compared with that in healthy individuals, particularly among clinically more severe and mobility-limited patients, whereas mental functioning appeared relatively preserved. Fatigue levels did not differ significantly between patients and controls. Height, fracture burden, history of orthopedic surgery, and alendronate use were associated with physical functioning, while fatigue severity was related to mental functioning. Given this study’s cross-sectional design, these findings should be interpreted as associations rather than causal effects and warrant confirmation in larger studies. Overall, our results highlight the need for comprehensive, patient-centered management strategies addressing not only skeletal outcomes but also fatigue and mental well-being in adults with OI.

## Figures and Tables

**Table 1:** Demographic and clinical characteristics of the study groups (OI types I, III, and IV) and healthy controls.

	OI Type I	OI Type III	OI Type IV	Control	*p* Value
**Age**	36(19–66)	25(18–37)	45(21–60)	38(21–55)	0.182
**Gender, female**	9(60)	2(40)	3(42.9)	14(51.9)	0.836
**Height, cm**	162(147–182)	124(110–148)	150(132–166)	169(150–198)	<0.001
**BMI**	23.8(17.9–29.6)	29.3(28.7–38.2)	26.7(21.2–30.1)	26.4(17.9–35.5)	0.003
**Education**					0.009
Primary	1(6.7)	-	4(57.1)	3(11.1)	
Secondary	5(33.3)	2(40)	3(42.9)	5(18.5)	
Tertiary	9(60)	3(60)	-	19(70.4)	
**Employed**	6(40)	2(40)	4(57.1)	23(85.2)	0.013
**Marital status, married**	10(66.7)	2(40)	4(57.1)	18(66.7)	0.676
**Smoking**	8(53.3)	0	4(57.1)	4(14.8)	0.004

Data are presented as *n* (%), median (min–max). Statistical significance was defined at *p* < 0.05.

**Table 2:** Frequency of physical examination findings and clinical characteristics across OI subtypes (types I, III, and IV).

	OI Type I	OI Type III	OI Type IV
**Upper limb deformity**	4(26.7)	1 (20)	1(14.3)
**Lower limb deformity**	1(6.7)	5(100)	3(42.9)
**Short stature**	10(66.7)	5(100)	6(85.7)
**Macrocephaly**	1(6.7)	1(20)	1(14.3)
**Pectus carinatum**	1(6.7)	3(60)	0
**Barrel chest**	4(26.7)	2(40)	2(28.6)
**Kyphosis**	2(13.3)	1(20)	4(57.1)
**Scoliosis**	5(33.3)	3(60)	5(71.4)
**Triangular face**	11(73.3)	5(100)	4(57.1)
**Colored sclera**	13(86.7)	4(80)	4(57.1)
**Hearing loss**	4(26.7)	1(20)	1(14.3)
**Dentinogenesis imperfecta**	10(66.7)	5(100)	4(57.1)
**Easy bruising**	6(40)	1(20)	2(28.6)
**Cardiovascular pathology**	1(6.7)	0	1(14.3)
**Independently ambulatory**	14(93.3)	0	7(100)
**Family history**	12(80)	4(80)	5(71.4)

Data are presented as *n* (%).

**Table 3:** Bone mineral density parameters and fracture-related characteristics by OI type.

		OI Type I	OI Type III	OI Type IV	*p* Value
**Lumbar total Z**		−2.5(−4.4/−0.20)	−3.0(−3.80/−0.90)	−3.3(−4.30/−2.10)	0.180
**Lumbar total BMD**		0.804(0.581–1.072)	0.715(0.619–0.983)	0.686(0.556–0.785)	0.343
**Femur total Z**		−0.9(−2.0–0.8)	−1.1(−1.2/−0.3)	−0.3(−1.6–0.1)	0.759
**Femur total BMD**		0.823(0.610–1.160)	0.833(0.810–0.897)	0.796(0.590–1.039)	0.910
Number of fractures *		4(1–10)	20(5–20)	5(3–15)	0.017
**Number of fractures**					0.003
**0–5**		7(46.7)	1(20)	4(57.1)	
**6–10**		8(53.3)	0	2(28.6)	
**>10**		0	4(80)	1(14.3)	
**First fracture age**		8(1–35)	0(0–5)	9(0–37)	0.058
**History of orthopedic procedures**	**History of orthopedic surgery**	6(40)	5(100)	5(71.4)	0.058
	**Fracture-related**	5(83.3)	1(20)	2(40)	0.149
	**Deformity-corrective/reconstructive**	0	2(40)	0	
	**Combined**	1(16.6)	2(40)	2(40)	
	**Childhood**	4(66.6)	2(40)	2(40)	0.123
	**Adulthood**	1(16.6)	0	3(60)	
	**Both periods**	1(16.6)	3(60)	0	

Data are presented as *n* (%), median (min–max). Statistical significance was defined at *p* < 0.05. * In patients with more than 15 fractures, the number was taken as 20. Z: Z score; BMD: bone mineral density.

**Table 4:** Use and duration of anti-osteoporotic therapies across OI subtypes (types I, III, and IV).

		OI Type I	OI Type III	OI Type IV	*p* Value
**Pamidronate**	***n*(%)**	4(26.7)	4(80)	0	0.013
	**Duration, mo**	18(12–48)	102(72–180)		0.011
**Zoledronic acid**	***n*(%)**	9(60)	5(100)	4(57.1)	0.260
	**Duration, mo**	42(18–84)	24(12–48)	24(12–108)	0.414
**Alendronate**	***n*(%)**	10(66.7)	3(60)	4(57.1)	1
	**Duration, mo**	66(1–138)	6(6–48)	42(12–60)	0.337
**Teriparatide**	***n*(%)**	2(13)	1(16)	2(28)	0.744
	**Duration, mo**	11(4–18)	12	18	NA
Other therapies, *n*(%) *		3(20)	1(20)	2(28.6)	NA
**Bisphosphonate duration, mo**		90(18–204)	141(18–216)	60(12–120)	0.178

* Other therapies: Ibandronate was used in two patients with OI type I and one patient with OI type IV; risedronate was used in one patient with OI type I and one patient with OI type III; denosumab was used in one patient with OI type IV. NA: not applicable due to small sample size.

**Table 5:** Health-related quality of life (SF-36) and fatigue (FSS) outcomes by OI type and controls.

	OI Type I	OI Type III	OI Type IV	Control	*p* Value
**PF**	90(55–100)	25(15–35)	60(30–95)	95(50–100)	<0.001
**RP**	75(0–100)	75(0–100)	0(0–75)	75(25–100)	0.023
**BP**	77.5(45–100)	70(20–87.5)	77.5(32.5–100)	80(22.5–100)	0.270
**GH**	65(25–100)	55(15–70)	60(40–85)	70(40–95)	0.049 *
**PCS**	50.14(17.03–68.59)	31.1(12.28–37.41)	33.13(11.91–54.07)	52.93(36.98–58.77)	<0.001
**PCS_HUN**	47.71(19.76–56.06)	24.17(7.73–42.48)	28.27(16.13–40.31)	45.68(25.31–55.8)	0.002
**RE**	66.7(0–100)	33.3(33.3–100)	66.7(33.3–100)	66.7(0–100)	0.867
**SF**	62.5(37.5–100)	62.5(37.5–87.5)	62.5(50–75)	87.5(0–100)	0.116
**MH**	72(12–96)	80(60–96)	76(52–84)	76(44–100)	0.675
**VT**	60(15–85)	60(50–95)	55(40–65)	60(35–90)	0.542
**MCS**	37.18(−13.88–59.69)	46.7(36.83–64.38)	44.81(25.14–60.98)	41.61(8.39–66.23)	0.337
**MCS_HUN**	52.16(−2.96–71.23)	62.95(42.8–77.43)	57.02(35.98–60.45)	55.88(25.59–74.51)	0.512
**FSS**	3.63 ± 1.78	2.82 ± 1.75	4.88 ± 1.72	3.53 ± 1.52	0.159
**FSS, group**					0.343
<4	9(60)	4(80)	2(28.6)	15(55.6)	
≥4	6(40)	1(20)	5(71.4)	12(44.4)	

Data are presented as *n* (%), median (min–max), or mean ± SD. Statistical significance was defined at *p* < 0.05. * No significant differences detected in the post hoc analysis. PF: physical functioning; RP: physical role limitations; BP: bodily pain; GH: general health; PCS: Physical Component Summary (for Hungary-based norms; PCS_HUN); RE: emotional role limitations; SF: social functioning; MH: mental health; VT: vitality; MCS: Mental Component Summary (for Hungary-based norms; MCS_HUN); FSS: Fatigue Severity Scale.

## Data Availability

The data presented in this study are available upon request from the corresponding author due to local ethical committee restrictions and data privacy.
